# The Role of Macrophages in HIV-1 Persistence and Pathogenesis

**DOI:** 10.3389/fmicb.2019.02828

**Published:** 2019-12-05

**Authors:** Zita Kruize, Neeltje A. Kootstra

**Affiliations:** Laboratory for Viral Immune Pathogenesis, Department of Experimental Immunology, Amsterdam UMC, Amsterdam Infection & Immunity Institute, University of Amsterdam, Amsterdam, Netherlands

**Keywords:** HIV-1, monocytes/macrophages, latency, pathogenesis, reservoir

## Abstract

Current antiretroviral therapy (ART) effectively suppresses Human Immunodeficiency Virus type 1 (HIV-1) in infected individuals. However, even long term ART does not eradicate HIV-1 infected cells and the virus persists in cellular reservoirs. Beside memory CD4^+^ T cells, cells of the myeloid lineage, especially macrophages, are believed to be an important sanctuary for HIV-1. Monocytes and macrophages are key players in the innate immune response to pathogens and are recruited to sites of infection and inflammation. Due to their long life span and ability to reside in virtually every tissue, macrophages have been proposed to play a critical role in the establishment and persistence of the HIV-1 reservoir. Current HIV-1 cure strategies mainly focus on the concept of “shock and kill” to purge the viral reservoir. This approach aims to reactivate viral protein production in latently infected cells, which subsequently are eliminated as a consequence of viral replication, or recognized and killed by the immune system. Macrophage susceptibility to HIV-1 infection is dependent on the local microenvironment, suggesting that molecular pathways directing differentiation and polarization are involved. Current latency reversing agents (LRA) are mainly designed to reactivate the HIV-1 provirus in CD4+ T cells, while their ability to abolish viral latency in macrophages is largely unknown. Moreover, the resistance of macrophages to HIV-1 mediated kill and the presence of infected macrophages in immune privileged regions including the central nervous system (CNS), may pose a barrier to elimination of infected cells by current “shock and kill” strategies. This review focusses on the role of monocytes/macrophages in HIV-1 persistence. We will discuss mechanisms of viral latency and persistence in monocytes/macrophages. Furthermore, the role of these cells in HIV-1 tissue distribution and pathogenesis will be discussed.

## Introduction

Antiretroviral therapy (ART) has dramatically improved the clinical outcome of Human Immunodeficiency Virus type 1 (HIV-1) infection. However, eradication of HIV-1 is not achieved due to persistence of a viral reservoir that harbors latent provirus that is reactivated upon discontinuation of ART. This latent viral reservoir is a major hurdle in curative treatment of HIV-1 infection and therefore new therapeutic approaches that aim to eliminate or reduce the viral reservoir are explored.

CD4+ T cells and cells from the monocyte/macrophage lineage are considered as the most important target cells for HIV-1, and play an important role in viral persistence and the formation of the viral reservoir. While the major viral reservoir in treated HIV-1 infection is comprised of CD4+ T cells, the distribution and characteristics of the monocyte/macrophage reservoir remain largely unknown.

Monocytes and macrophages are part of the innate immune system and respond to different signals which will direct macrophage differentiation, polarization and function. Monocytes/macrophages sense and clear pathogens, serve as antigen presenting cells priming the adaptive immune response, are involved in both pro- and anti-inflammatory responses, and tissue repair. Macrophages are found in all lymphoid as well as non-lymphoid tissues and their origin was originally described to be dependent on circulating monocytes, which would replenish the tissue-resident macrophage pool if needed. Recent studies in mice showed that the majority of tissue-resident macrophages are embryonically derived and have been implicated to be self-renewing, with the exception of the mucosal/border tissues like the intestine, the dermis and the heart, where bone marrow-derived circulating monocytes constantly replenish the aging tissue-resident macrophage pool (Ginhoux et al., 2010; Schulz et al., 2012; Hashimoto et al., 2013; Yona et al., 2013; Bain et al., 2014; Hoeffel et al., 2015; Mass et al., 2016). The proportion between the pool of tissue-resident macrophages and the pool of monocytes-derived macrophages vary during a state of homeostasis or inflammation (Wang and Kubes, 2016).

Upon differentiation of monocytes into macrophages, these cells become susceptible to HIV-1 infection and due to their ability to migrate into tissues, they contribute to the spread of viral infection to nearly every tissue of the body including gut, semen, lung, gut-associated lymphoid tissue, brain, liver, urethra, and lymph nodes (Zhang et al., 1998; Mayer et al., 1999; Lambotte et al., 2005; Guadalupe et al., 2006; Poles et al., 2006; Chun et al., 2008; Zalar et al., 2010; Deleage et al., 2011; Abbas et al., 2014; Yukl et al., 2014; Cribbs et al., 2015; Hansen et al., 2016; Rose et al., 2016; Kandathil et al., 2018; Tso et al., 2018; Ganor et al., 2019; Ko et al., 2019). Furthermore, macrophages are considered long lived, resistant to virally induced cytopathic effects, and can reside in anatomical sanctuaries with restricted penetration of ART, which will support viral persistent even during ART (Ho et al., 1986; Nicholson et al., 1986; Lafeuillade et al., 1998; Solas et al., 2003; Estes et al., 2017; Clayton et al., 2018).

In this review we will discuss the role of monocytes/macrophages in HIV-1 pathogenesis, HIV-1 persistence and viral reservoir.

## Monocytes and Macrophages in HIV-1 Pathogenesis

### HIV-1 Transmission

Human Immunodeficiency Virus type 1 enters the body mostly through mucosal surfaces of the genital or gastrointestinal tract. The underlying lamina propria is populated with high populations of lymphocytes and macrophages, which express CD4 in combination with the coreceptor C-C chemokine receptor type 5 (CCR5) and serve as targets for HIV-1 infection. Although the CD4+ T cells in the lamina propria are considered the major target for HIV-1 (Zhang et al., 1999; Greenhead et al., 2000; Gupta et al., 2002; Guadalupe et al., 2003; Brenchley et al., 2004; Hladik et al., 2007), infection of macrophages located in the lamina propria of the intestinal, penile urethral and vaginal mucosa has been described (Shen et al., 2009; Zalar et al., 2010; Ganor et al., 2013; Josefsson et al., 2013; Yukl et al., 2014). This confirms a role for macrophages during the early phases of infection, however, it remains under debate whether these mucosal macrophages support HIV-1 replication and spread the infection. Indeed, HIV-1 RNA has been demonstrated to be present in macrophages located in the vagina (Shen et al., 2009), but not in the gut, indicating that local environmental signals required for efficient viral replication in macrophages can be lacking in local mucosal tissues under homeostatic conditions.

### Monocytes in HIV-1 Infection

Human Immunodeficiency Virus type 1 infection is characterized by high immune activation and inflammation, caused by high levels of HIV-1 replication, bacterial translocation, coinfection with other viruses (e.g., CMV, HCV, HBV) and, immune dysregulation (Brenchley and Douek, 2008; Deeks, 2011). ART strongly reduces HIV-1 associated inflammation and immune activation, however, residual immune activation and inflammation is still observed even in effectively treated individuals (Brenchley and Douek, 2008; Deeks, 2011). During HIV-1 infection, increased activation and differentiation of peripheral blood monocytes is also observed (Allen et al., 1990; Thieblemont et al., 1995; Pulliam et al., 1997; Ancuta et al., 2008; Hearps et al., 2012; Williams et al., 2012; Westhorpe et al., 2014; Booiman et al., 2017), which may affect their susceptibility to HIV-1 infection and their ability to migrate into the tissues, thus contributing to HIV-1 pathogenesis.

In the peripheral blood, three monocyte subpopulations can be distinguished based on the expression of the LPS receptor CD14 and the FcγIII receptor CD16. The majority of the peripheral blood monocytes (>85%) are classical monocytes that express high levels of CD14 (CD14++CD16-), whereas CD16 is co-expressed on the intermediate (CD14++CD16+) and non-classical monocytes (CD14+CD16+) (Passlick et al., 1989; Ziegler-Heitbrock et al., 2010; Wong et al., 2011). Murine studies and more recently a humanized mouse study showed that monocyte precursors differentiate first into classical monocytes in bone marrow for a maturation phase of ∼ 38 h. This classical monocyte population is retained in the bone marrow and can respond to acute systemic inflammation. Most classical monocytes remain in the circulation for approximately 1–3 day before migration into the tissues. However, a small proportion matures further into intermediate monocytes within the circulation and most of these cells finally mature into non-classical monocytes before leaving the circulation (Sunderkotter et al., 2004; Varol et al., 2007; Yona et al., 2013; Gamrekelashvili et al., 2016; Patel et al., 2017). In HIV-1 infected individuals, an expansion of CD16 expressing monocytes or intermediate monocytes (CD14+CD16+) is evident during all phases of infection, especially in viremic individuals, which is normalized after initiation of ART in at least part of the patients (Allen et al., 1990; Thieblemont et al., 1995; Pulliam et al., 1997; Ancuta et al., 2008; Hearps et al., 2012; Williams et al., 2012; Westhorpe et al., 2014; Booiman et al., 2017). Moreover, monocytes express increased levels of activation markers (e.g., CD163, HLA-DR, CD69), T-cell costimulatory molecules (e.g., CD40 and CD86), adhesion molecules (e.g., CD11b, CD11c, CD91, CX3CR1), and chemokine receptors (e.g., C-C chemokine receptor type 2 (CCR2), CCR5), which is also observed during ART (Pulliam et al., 1997; Ancuta et al., 2008; Hearps et al., 2012; Westhorpe et al., 2014; Booiman et al., 2017).

In the peripheral blood, a small population (<0.1%) of the monocytes harbor replication competent HIV-1 provirus (Furtado et al., 1999; Crowe and Sonza, 2000; Sonza et al., 2001; Shiramizu et al., 2005; Ellery et al., 2007). While classical monocytes are relatively resistant to HIV-1 infection most likely due to low CCR5 expression, CD16+ or intermediate monocytes express higher levels of CCR5 and can be infected by HIV-1 (Ellery et al., 2007; Ancuta et al., 2008). Indeed, CD16+ or intermediate monocytes have been demonstrated to harbor proviral DNA in untreated as well as treated HIV-1 infection (Shiramizu et al., 2005; Jaworowski et al., 2006, 2007; Ellery et al., 2007).

### Tissue Macrophages as HIV-1 Reservoirs

**FIGURE 1:**
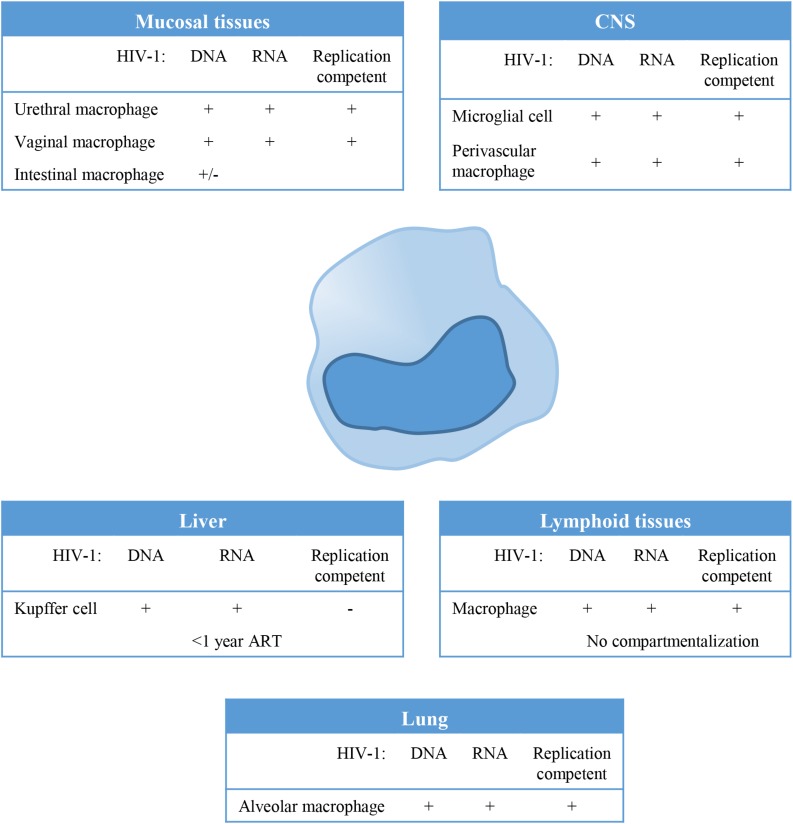
Tissue distribution and anatomical sanctuaries of latently infected macrophages. Macrophages can be found in all lymphoid as well as non-lymphoid tissues and could therefore serve as a tissue reservoir for Human Immunodeficiency Virus type 1(HIV-1). This figure summarizes findings on HIV-1 latency in monocytes/macrophages *in vivo*, showing anatomical sanctuaries, the presence of HV-1 DNA and RNA and whether the virus was replication competent or not.

Macrophages can be found in all lymphoid as well as non-lymphoid tissues and could therefore serve as a tissue reservoir for HIV-1 (Figure 1). In the lamina propria of mucosal tissues like the gastro intestinal tract, penile urethra and vagina, HIV-1 infection of local macrophages has been demonstrated in tissues from healthy individuals *in vitro* (Shen et al., 2009; Ganor et al., 2013), and *in vivo* in HIV-1 infected untreated (Josefsson et al., 2013) and ART treated individuals (Zalar et al., 2010; Josefsson et al., 2013; Yukl et al., 2014; Ganor et al., 2019). Although, HIV-1 DNA has been readily detected in these mucosal tissues, viral RNA has been demonstrated only in vaginal and urethral macrophages (Shen et al., 2009; Ganor et al., 2019). Moreover, replication competent HIV-1 could be isolated from urethral macrophages (Ganor et al., 2019). In contrast, HIV-1 proviral integration could not be detected in macrophages from colon of ART treated HIV-1 infected individuals (Cattin et al., 2019), and therefore it seems unlikely that these cells contribute to the replication competent HIV-1 reservoir (Shen et al., 2011).

In lymphoid tissues like spleen and lymph node, CD4+ T cells (resting memory cells) are the predominant reservoir, however, latently infected resident macrophages have been detected at low frequencies in lymph nodes (Embretson et al., 1993; Orenstein et al., 1997). HIV-1 residing in lymphoid tissues does not genetically differ from variants circulating in the blood, which suggest that there is no compartmentalization of HIV-1 in lymphoid tissue (Ball et al., 1994; van’t Wout et al., 1998). However, genetic diversity of HIV-1 and independent viral evolution in monocytes and CD4+ T cells during the course of infection in treated and untreated patients suggests cellular compartmentalization (Fulcher et al., 2004; Llewellyn et al., 2006).

Tissue resident macrophages like Kupffer cells in the liver have been demonstrated to be susceptible to HIV-1 infection, and persistence of HIV-1 in Kupffer cells has been demonstrated in HIV-1 infected individuals on ART (Schmitt et al., 1990; Hufert et al., 1993; Mosoian et al., 2017; Kandathil et al., 2018). However, virus particles produced by Kupffer cells could not be propagated *in vitro* using CEMx174 cells (Kandathil et al., 2018). Phylogenetic analysis of the viral quasi-species showed distinct clustering of viral variants in the liver, evident of compartmentalization of HIV-1 in the liver (Penton and Blackard, 2014). Also alveolar macrophages in the lung have been identified as HIV-1 targets, and HIV-1 persistence as determined by the presence of HIV-1 DNA and HIV-1 RNA has been demonstrated in patients, even during effective ART (Sierra-Madero et al., 1994; Jambo et al., 2014; Cribbs et al., 2015). Analysis of the HIV quasi-species also suggested compartmentalization of macrophage tropic HIV-1 variants in the lung (van’t Wout et al., 1998).

The central nervous system (CNS) has been identified as an important viral reservoir. HIV-1 enters the CNS through trafficking of infected cells across the blood brain barrier (BBB) which occurs already shortly after primary infection. Indeed, HIV-1 associated monocyte activation increases migratory abilities of peripheral monocytes by the upregulation of adhesion molecules and chemokine receptors, in response to for instance the monocyte chemoattractant protein 1 (MCP1) (Ancuta et al., 2004; Williams et al., 2013, 2014). In the CNS, HIV-1 persists predominantly in resident macrophages, like microglial cells and perivascular macrophages (Stoler et al., 1986; Wiley et al., 1986; Neuen-Jacob et al., 1993; Fischer-Smith et al., 2001; Cosenza et al., 2002; Ko et al., 2019). Genetic analysis of the viral quasi-species revealed compartmentalized viral replication in the CNS (Korber et al., 1994; van’t Wout et al., 1998; Schnell et al., 2009).

Although HIV-1 infection of macrophages has been demonstrated in different tissue, their role as a replication-competent reservoir remains under debate (Figure 1). Macrophages are terminally differentiated and their contribution to the viral reservoir is mainly dependent on their lifespan, whereas latently infected resting CD4+ memory T cells which are also long lived cells, contribute to the viral reservoir through homeostatic or antigen specific proliferation. However, recent reports have clearly demonstrated that macrophages can be infected *in vivo* and contribute to the viral reservoir (Figure 1; Micci et al., 2014; Clayton et al., 2017; DiNapoli et al., 2017). In SHIV infected rhesus macaques, *in vivo* viral replication was sustained by tissue macrophages upon CD4 T cell depletion (Igarashi et al., 2001). Moreover, HIV-1 persistence in macrophages was confirmed in HIV-1 infected humanized myeloid only mice in which viral rebound was observed in 33% of the animals following treatment interruption (Honeycutt et al., 2017).

### Macrophages in HIV-1 Related Comorbidities

Monocytes and macrophages play a crucial role in several HIV-1 related comorbidities including neurological disorders, atherosclerosis and cardiovascular disease (CVD). HIV-1 associated neurocognitive disorders (HAND) describe a broad range of neurocognitive disorders from asymptomatic neurocognitive impairment, mild neurocognitive disorders (MND), and HIV-1 associated dementia (HAD). Although the use of ART has dramatically reduced development of HAD, milder forms of HAND are still highly prevalent amongst effectively treated HIV-1 infected individuals (Robertson et al., 2007; Heaton et al., 2010; Simioni et al., 2010). In HIV-1 infection, activated peripheral blood monocytes have increased migratory ability (Ancuta et al., 2004; Williams et al., 2013, 2014) and can cross the BBB, and therefore it is generally believed that HIV-1 infected peripheral monocytes are the main source of HIV-1 infection in the brain. Increased monocyte activation, expansion of the activated monocyte population as well as the level of HIV-1 DNA positive monocytes (CD14+/CD16+) have been associated with HIV-1 associated neuroinflammation and HAND (Pulliam et al., 1997, 2004; Shiramizu et al., 2005; Williams et al., 2014; Booiman et al., 2017). The ongoing neuroinflammation caused by monocyte activation and migration, is considered a strong mediator of neuronal damage in HAND. Moreover, persistent HIV-1 replication in resident macrophages and microglial cells of the brain, contributes to the ongoing neuroinflammation and neuronal damage. In particular the viral envelope protein gp120 (Giulian et al., 1993; Meucci et al., 1998; Kaul and Lipton, 1999; Ohagen et al., 1999), the HIV-1 accessory protein Vpr (Piller et al., 1998; Patel et al., 2000) and the HIV-1 transcriptional activator Tat (Liu et al., 2000; Park et al., 2001; Andras et al., 2003; Song et al., 2003) have been identified to contribute directly or indirectly to neurotoxicity.

HIV-1 associated inflammation and immune activation, both risk factors in the onset of atherosclerosis and CVD, likely contribute to the increased risk of CVD in the HIV-1 infected population (Nou et al., 2016; Triant and Grinspoon, 2017). In early stages of atherosclerosis development, hyperlipidemia [(cholesterol, triglyceride, and low-density lipoprotein (LDL)] promotes the retention and uptake of LDL in the sub-endothelial space (Fogelstrand and Boren, 2012), which results in activation of endothelial cells and smooth muscle cells by the upregulation of adhesion molecules [intercellular adhesion molecule 1 (ICAM-1) and vascular cell adhesion protein 1 (VCAM-1)] and secretion of chemokines [MCP-1 and macrophage colony-stimulating factor (M-CSF)] in the arterial wall (Frostegard et al., 1991). Cells of the immune system expressing the MCP-1 receptor CCR2, are recruited and can infiltrate the lesion (Frostegard et al., 1991). During HIV-1 infection, circulating activated monocytes express increased levels of CCR2, and have an increased ability to migrate in response to MCP1 into the arterial wall (Ancuta et al., 2004; Williams et al., 2013, 2014). These infiltrating monocytes further increase arterial inflammation by cytokine production [interleukin 1ß (IL-1ß) and IL-6] and uptake of (modified) lipoproteins, thereby accelerating atherosclerosis and CVD progression (Hansson, 2005). Indeed, it has been shown that aortic inflammation as measured by ^18^fluorodeoxyglucose uptake (^18^F-fluorodexoyglucose Positron Emission Tomography – Computed Tomography: ^18^F-FDG PET-CT) was increased in effectively treated HIV-1 infected patients to a degree resembling that of uninfected individuals with known CVD. Furthermore, ^18^F-FDG PET-CT activity was associated with serum levels of soluble CD163 (sCD163), a marker of monocyte/macrophage activation (Subramanian et al., 2012; Tawakol et al., 2017), clearly indicating a role of these cells in accelerated onset of atherosclerosis and CVD in HIV-1 infection.

## Mechanisms of HIV-1 Latency

### Macrophage Differentiation and Polarization

Human Immunodeficiency Virus type 1 infection in macrophages and their role in the viral reservoir are difficult to study *in vivo* and therefore *in vitro* culture models have been developed. In these models, monocytes are isolated from peripheral blood and differentiated in an *in vitro* culture system toward monocyte derived macrophages. In addition, different stimuli or cytokines can be added to the cultures in order to obtain phenotypically and functionally distinct macrophages. An advantage of this *in vitro* culture system is that large number of cells are available for analysis and macrophage activation, differentiation, polarization and function can easily be directed. A major disadvantage is that conditions in the culture system are not representative of the local environment in the different tissues, as tissue resident macrophages continuously respond to local signals influencing their function and phenotype resulting in a heterogeneous resident macrophage population. Recently, macrophages isolated from tissues of HIV-1 infected individuals have provided a better insight in the *in vivo* relevance and the state of the viral reservoir (latent, activated) (Zalar et al., 2010; Yukl et al., 2014; Cribbs et al., 2015; Kandathil et al., 2018; Ganor et al., 2019; Ko et al., 2019). However, these materials are difficult to obtain and invasive for the patient.

*In vitro*, monocytes become highly susceptible to HIV-1 infection upon differentiation into macrophages (Rich et al., 1992; Schuitemaker et al., 1992; Sonza et al., 1996; Peng et al., 2007; Wang et al., 2009; Cobos Jimenez et al., 2012). Macrophages respond to different stimuli in their environment which will shape their physiology and function. Interferon-γ (IFN-γ) and tumor necrosis factor-α (TNF-α) polarize macrophages into an M1 or pro-inflammatory phenotype (Mosser, 2003). M2 or alternatively activated macrophages are generated upon exposure to IL-4 or IL-13 (M2a), immunoglobulin G (IgG) immune complexes and toll-like receptor (TLR) agonists (M2b) or IL-10 or glucocorticoids (M2c) (Mosser, 2003; Mantovani et al., 2004; Mosser and Edwards, 2008; Martinez et al., 2009). Polarization toward a M2a phenotype results in resistance to HIV-1 infection due to restrictions at the level of reverse transcription (Schuitemaker et al., 1992; Montaner et al., 1997; Wang et al., 1998; Cobos Jimenez et al., 2012), whereas polarization toward M1 or M2c inhibits viral replication at a transcriptional or post-transcriptional level (Kootstra et al., 1994; Montaner et al., 1994; Naif et al., 1996; Perez-Bercoff et al., 2003; David et al., 2006; Cassol et al., 2009, 2010; Cobos Jimenez et al., 2012). This data suggests that polarizing signals can induce viral latency in macrophages at both a pre- or post-integration level (Table 1).

**TABLE 1:** Cellular/viral factors involved in pre- and post-integration latency.

**Latency level**	**Block in viral replication cycle**	**Cellular/viral factor involved**	**Cell type**	**References**
**Pre-integration**	Reverse transcription	APOBEC3A	Monocytes/macrophages, monocytic cell line	Peng et al., 2007; Miyagi et al., 2008; Berger G. et al., 2011
		Low dNTPs pool	Macrophages, monocytic cell line;	Kootstra et al., 2000; Lahouassa et al., 2012
			CD4+ T cells	Korin and Zack, 1999
		SAMHD1	Macrophages, monocytic cell lines;	Berger A. et al., 2011; Hrecka et al., 2011; Laguette et al., 2011; Lahouassa et al., 2012
			CD4+ T cells	Baldauf et al., 2012; Descours et al., 2012
		PAF1	Monocytes/macrophages;	Liu et al., 2011; Tyagi and Kashanchi, 2012
			CD4+ T cells	Liu et al., 2011; Tyagi and Kashanchi, 2012
	Nuclear import	MX2	Macrophages, monocytic cell lines;	Goujon et al., 2013; Kane et al., 2013; Wang et al., 2017; Buffone et al., 2019
			CD4+ T cells	Goujon et al., 2013; Kane et al., 2013
**Post-integration**	Transcriptional regulation	SP1, CTIP2, SUV39H1, HDACs, c-myc, and HP1 in complex	Monocytes/macrophages, monocytic cell lines	Rohr et al., 2003; Marban et al., 2005, 2007; du Chene et al., 2007; Desplats et al., 2013; Gray et al., 2016
			CD4+ T cells, T cell lines	du Chene et al., 2007; Jiang et al., 2007
		HDACs, CTIP2, YY1, CBF-1 and LSF in complex	Monocytes/macrophages, monocytic cell line	Buescher et al., 2009
			T cell lines	Margolis et al., 1994; Coull et al., 2000; He and Margolis, 2002; Tyagi and Karn, 2007
		CTIP2, LSD1, and hSET1/WDR5 in complex	Monocytes/macrophages monocytic cell line	Le Douce et al., 2012
		DYRK1A and NFAT	Macrophages	Bol et al., 2011
			CD4+ T cells, T cell line	Booiman et al., 2015; Heffern et al., 2019
		TCF-4, β-catenin, and SMAR1 complex	Monocytes/macrophages, monocytic cell line, PBMCs	Kumar et al., 2008; Henderson et al., 2012; Aljawai et al., 2014
		CTIP2, Tat, and P-TEFb	Monocytes/macrophages, monocytic T cells	Herrmann et al., 1998; Liou et al., 2002; Rice and Herrmann, 2003; Cherrier et al., 2013
			CD4+ T cells	Herrmann et al., 1998; Rice and Herrmann, 2003
	Transcription/Translation	miRNAs (miR-17/92, miR-28, miR-150, miR-223 and miR-382)	Monocytes/macrophages, monocytic cell line	Sung and Rice, 2009; Wang et al., 2009; Lodge et al., 2017
			CD4+ T cells, T cell line, PBMCs	Huang et al., 2007; Triboulet et al., 2007
	Nuclear export	MATR3, PSF and Rev	CD4+ T cells	Sarracino et al., 2018
	Translation	G3BP1	Monocytes/macrophages	Cobos Jimenez et al., 2015
			CD4+ T cell	Cobos Jimenez et al., 2015

### Mechanisms of Pre-integration Latency

Integration of HIV-1 proviral DNA into the host genome is essential for viral replication. Upon entry into the cell, HIV-1 RNA is reverse transcribed, and subsequently the pre-integration complex (PIC), consisting of viral proteins (integrase, matrix, Vpr, capsid) and double stranded DNA (dsDNA) is assembled (Greene and Peterlin, 2002). The PIC is transported into the nucleus and where the proviral DNA integrates into the host cell genome. Pre-integration latency results when the viral replication cycle is partially or completely blocked at steps prior to integration (Table 1). Pre-integration latency is frequently observed in CD4+ T cells but was assumed not to be clinically relevant, because *in vitro* studies demonstrated that the unintegrated proviral DNA only persists for 1 day in these cells (Pierson et al., 2002; Zhou et al., 2005). However, in patients treated with ART, including an integrase inhibitor, unintegrated HIV-1 DNA subspecies where shown to persist in CD4+ T cells and can potentially integrate when integrase inhibitor medication is discontinued (Murray et al., 2014). Moreover, metabolically active macrophages can contain large quantities of unintegrated viral DNA, which remains stable and biologically active in non-dividing macrophages for up to 2 months (Gillim-Ross et al., 2005; Kelly et al., 2008). Therefore, unintegrated HIV-1 DNA in macrophages may contribute to the viral reservoir in HIV-1 pathogenesis. Indeed, some studies have reported detection of unintegrated HIV-1 DNA in macrophages from patients, mostly in the brain (Pang et al., 1990; Teo et al., 1997). In monocytes/macrophages pre-integration latency could be a consequence of poor reverse transcription efficiency due to a reduced deoxynucleotide triphosphates (dNTP) pool or inhibition of nuclear transport of the PIC as a consequence of low adenosine triphosphate (ATP) levels (Bukrinsky et al., 1992; Kootstra et al., 2000; Kennedy et al., 2010; Lahouassa et al., 2012). Several cellular factors have been implicated to play a role in the observed restriction in monocytes/macrophages: apolipoprotein B mRNA editing enzyme catalytic subunit 3G (APOBEC3G) and subunit 3A (APOBEC3A), a cellular deoxycytidine deaminases which induces G to A hypermutation and degradation of the HIV-1 genome (Peng et al., 2007; Miyagi et al., 2008; Berger G. et al., 2011); Sterile α-motif/histidine-aspartate domain-containing protein 1 (SAMHD1) which hydrolyses dNTPs into their precursors (nucleosides and triphosphates), in turn reducing the dNTPs pool and thereby limiting the reverse transcriptase activity (Berger A. et al., 2011; Hrecka et al., 2011; Laguette et al., 2011; Lahouassa et al., 2012); Myxovirus-resistance protein 2 (MX2) which inhibits HIV-1 replication post entry by hindering the nuclear accumulation and integration of proviral DNA into host chromatin (Goujon et al., 2013; Kane et al., 2013; Wang et al., 2017; Buffone et al., 2019); and members of the RNA polymerase II-associated factor 1 (PAF1) family, which are expressed in monocytes/macrophages and repress HIV-1 reverse transcription and proviral DNA integration (Liu et al., 2011; Tyagi and Kashanchi, 2012).

### Mechanisms of Post-integration Latency

Post-integration latency, which is established after integration of the HIV-1 proviral DNA into the host chromatin, is in contrast to pre-integration latency very stable and only limited by the lifespan of the infected cell. Mechanisms underlying HIV-1 latency *in vivo* are incompletely understood, however, it is a multifactorial phenomenon. Post-integration viral latency may be maintained at a transcriptional or a post-transcriptional level inhibiting HIV-1 protein production and virus formation. Several mechanisms acting at transcriptional and post-transcriptional level inducing HIV-1 latency in CD4+ T cells are well described. However, it is unknown whether these mechanism are also effective in cells of the monocyte/macrophage lineage. Nevertheless, several mechanisms inducing HIV-1 post-integration latency have been described in monocytes/macrophages, including chromatin environment, the absence of transcriptional activation, presence of transcriptional repressors and host antiviral processes. However, not all of these mechanisms are specific for post-integration latency in monocytes/macrophages (Table 1).

#### Site of Integration and Chromatin Remodeling

The host chromatin is organized into heterochromatin which is densely packed and transcriptionally silent and euchromatin which is loosely packed and transcriptionally active (Demeret et al., 2001). After nuclear import a transcriptional co-activator, lens epithelium-derived growth factor (LEDGF)/p75, which interacts with HIV-1 integrase (Vandegraaff et al., 2006; Meehan et al., 2009), targets the PIC mainly to intronic regions of actively transcribed genes (Schroder et al., 2002; Lewinski et al., 2006). The majority of silent HIV-1 provirus is integrated into the euchromatin, which seems paradoxical considering the fact that euchromatin is transcriptionally active (Han et al., 2004). Different mechanisms of transcriptional interference (e.g., steric hindrance, promotor occlusion and enhancer trapping) have been suggested as possible explanation for integrated proviral DNA expression suppression (Lassen et al., 2004; Bisgrove et al., 2005; Taube and Peterlin, 2013). Most of the data on HIV-1 integration and latency has been derived from CD4+ T cells. However, the integration of HIV-1 proviruses has also been studied in primary macrophages. Barr et al., for instance, studied HIV-1 DNA integration sites in primary macrophages by sequencing 754 unique integration sites and found that, similar to CD4+ T cells, HIV-1 also integrates preferentially into the transcriptionally active regions in macrophages (Mack et al., 2003; Barr et al., 2006; Wellensiek et al., 2009). In CD4+ T cells transcription gene silencing is the most favored mechanism for the establishment and maintenance of HIV-1 latency, whether this is also the case in monocytes/macrophages still has to be determined. However, it is well established that chromatin organization and reorganization influences the gene expression and that HIV-1 proviral DNA follows the same rules that apply to host genes. The role of histone H3 lysine 9 trimethylation (H3K9me3) in heterochromatin formation and the transcriptional silencing of integrated HIV-1 has been described (Grewal and Moazed, 2003; Stewart et al., 2005; du Chene et al., 2007; Marban et al., 2007). In addition, there are two nucleosomes, nuc-0 and nuc-1, that interact with the HIV-1 promoter irrespective of the HIV-1 integration site in the host genome. HIV-1 gene transcription from proviral DNA is only possible with the displacement of nuc-1 (Verdin et al., 1993). These studies were performed in the monocytic U1 cell line and collectively suggest that chromatin remodeling is an essential mechanism of HIV-1 latency establishment and regulation in monocytes/macrophages, however, these observations have not yet been confirmed in primary monocytes/macrophages.

#### Transcriptional Regulation

The integrated HIV-1 proviral DNA is flanked by the long terminal repeats (LTR). The 5′LTR has binding sites for several transcription factors including specificity protein 1 (Sp1), activator protein 1 (AP1), nuclear factor of activated T-cells (NFAT), nuclear factor-κB (NF-κB), c-myc, chicken ovalbumin upstream promoter (COUP), upstream stimulatory factor (USF), CCAAT box transcription factor/nuclear factor 1 (CTF/NF1), T cell factor 1α (TCF-1α) and the glucocorticoid receptor (Verdin et al., 1993). These transcription factors act together to regulate HIV-1 proviral DNA expression (Table 1). Sp1, for instance, recruits c-myc to the 5′LTR of proviral DNA, which in turn recruits histone deacetylase 1 (HDAC1). HDAC1 then induces chromatin remodeling, which results in the suppression of HIV-1 gene expression (Jiang et al., 2007; Gray et al., 2016). In addition, HDACs were also shown to be recruited to the proviral promoter by COUP transcription factor interacting protein 2 (CTIP2), Ying Yang 1 (YY1), C-promoter binding factor-1 (CBF-1) and Late SV40 Factor (LSF), thereby promoting the establishment of latency (Coull et al., 2000; He and Margolis, 2002; Tyagi and Karn, 2007; Buescher et al., 2009; Le Douce et al., 2010). Similar results were observed in microglial cells, where CTIP2 has been shown to recruit both HDAC1 and HDAC2 to the 5′LTR of HIV-1 proviral DNA (Marban et al., 2007). Moreover, CTIP2 also interacts with suppressor of variegation 3–9 homolog 1 (SUV39H1), a methyl transferase for H3K9me3 which subsequently promotes the recruitment of heterochromatin protein 1 (HP1) leading to local heterochromatization and induction of viral latency (Rohr et al., 2003; Marban et al., 2005; du Chene et al., 2007). The role of CTIP2 was further confirmed by Desplats et al. (2013) who analyzed the expression of CTIP2 in postmortem brain tissue of HIV-1 infected patients with HIV-1 encephalitis (HIV-1 DNA and RNA positive) and HIV-1 positive patients with latent HIV-1 (HIV-1 DNA positive and HIV-1 RNA negative) and no detectable HIV-1 (HIV-1 DNA negative) in the CNS. Higher amounts of CTIP2 were observed in the HIV-1 patients with latent HIV-1 in the CNS as compared to the other groups. Notably, CTIP2 was detected in microglial cells of patients with latent HIV-1, indicating that CTIP2 may play an important role in the regulation of viral latency in microglial cells (Desplats et al., 2013). Furthermore, lysine-specific demethylase 1 (LSD1) was found to regulate HIV-1 gene expression in a synergistic way with CTIP2 in microglial cells (Le Douce et al., 2012). LSD1 also assists in the recruitment of CTIP2 and human SET1/WD40-repeat protein 5 (hSET1/WDR5) at the Sp-1 binding sites of the HIV-1 proximal promoter, resulting in increased H3K4 trimethylation (H3K4me3), which in turn represses viral gene expression (Le Douce et al., 2012). This property of LSD1 seems to be highly specific for cells of the monocyte/macrophage lineage.

Recently, the host factor dual specificity tyrosine-phosphorylation-regulated kinase 1A (DYRK1A) was shown to control HIV-1 replication by regulating HIV-1 provirus transcription in macrophages and CD4+ T cells (Bol et al., 2011; Booiman et al., 2015). DYRK1A was shown to regulate HIV-1 transcription via phosphorylation of the NFAT, thereby promoting NFAT translocation from the nucleus to the cytoplasm resulting in decreased viral transcription and latency. Chemical inhibition of DYRK1A resulted in an increased NFAT binding to the viral LTR and reactivating latent provirus (Bol et al., 2011; Booiman et al., 2015).

Furthermore, it has been reported the 5′LTR of the HIV-1 provirus contains multiple T cell factor 4 (TCF-4) binding sites. TCF-4 interacts with β-catenin and the scaffold matrix attachment region-binding protein 1 (SMAR1) at the 5′LTR and this complex has been shown to repress HIV-1 proviral gene expression in different cell types including lymphocytes and astrocytes (Kumar et al., 2008; Henderson et al., 2012). β-catenin/TCF4 signaling has also been demonstrated to regulate HIV-1 replication in cells of the monocyte/macrophage lineage (Aljawai et al., 2014), which makes it plausible that these proteins also play a role in viral latency in these cells (Kumar et al., 2008).

Apart from cellular factors, HIV-1 latency is also influenced by viral factors. HIV-1 replication requires the viral *trans-*activator protein Tat for efficient transcription and virus production. In the absence of Tat, only low level of HIV-1 transcription of mostly short abortive transcripts is observed (Karn, 2011). These short RNA transcripts contain a *trans-*activation response (TAR) element which is the binding site for the Tat protein (Berkhout et al., 1989). The positive transcription elongation factor (P-TEFb) is a critical cellular cofactor of Tat, that favors the generation of complete transcripts from HIV-1 proviral DNA (Price, 2000; Karn, 2011). P-TEFb is composed of a catalytic subunit, cyclin-dependent kinase 9 (CDK9), and a regulatory subunit, cyclin T1 (CycT1) (Herrmann et al., 1998; Rice and Herrmann, 2003; Bres et al., 2008). Tat binds to the TAR region of the RNA transcript and directs P-TEFb to the RNA polymerase II resulting in transcription of full-length HIV-1 RNA (Berkhout et al., 1989; Karn, 2011; Cherrier et al., 2013). The CDK2 can phosphorylate Ser90 on CDK9, thereby assisting in HIV-1 transcription (Ammosova et al., 2005; Breuer et al., 2012). Monocytes express very low levels of CycT1, which transiently increases during differentiation into macrophages. In contrast CDK9 expression levels remain constant in monocytes and macrophages (Liou et al., 2002). The low levels of CycT1 in monocytes result in low functional levels of P-TEFb, resulting in low HIV-1 transcription and thus viral latency in monocytes (Liou et al., 2002).

#### Post-transcriptional Regulation

Human Immunodeficiency Virus type 1 latency can also be regulated at a post-transcriptional level (Table 1). miRNAs are small single stranded non-coding RNAs that can regulate host gene expression at a post-transcriptional level. miRNAs have also been reported to influence HIV-1 gene expression (Huang et al., 2007; Sung and Rice, 2009; Haasnoot and Berkhout, 2011; Narayanan et al., 2011; Lodge et al., 2017). For instance, the miRNA cluster miR-17/92 is actively inhibited by HIV-1 to support efficient replication in different cell types including lymphocytes, monocytes and macrophages (Triboulet et al., 2007). Furthermore, miR-28; miR-150; miR-223; and miR-382 have been demonstrated to target HIV-1 and play a role in HIV-1 susceptibility of monocytes and macrophages. Inhibition of these miRNAs in monocytes results in increased HIV-1 replication, while increased expression of these miRNAs in macrophages decrease of HIV-1 replication (Huang et al., 2007; Wang et al., 2009). These data indicate that miRNAs can play an important role in viral latency in macrophages.

RNA binding host proteins like GTPase-activating protein-(SH3 domain)-binding protein 1 (G3BP1) and the matrix-associated RNA binding protein Matrin 3 (MATR3), have been shown to play a role in the post-transcriptional regulation of HIV-1 latency. G3BP1 is a single-strand-specific endonuclease that binds mRNA transcripts at the 3′UTR inside stress granules to control mRNA translation during cellular stress (Tourriere et al., 2001; Ortega et al., 2010). G3BP1 was shown to interact with HIV-1 mRNA transcripts thus preventing viral protein translation and restricting HIV-1 replication in T cells and macrophages (Cobos Jimenez et al., 2015). Resting T cells and IFNγ and TNFα polarized macrophages, express high levels of G3BP1 indicating a role of this host factor in HIV-1 latency (Cobos Jimenez et al., 2015). MATR3 is a nuclear matrix protein that has the ability to bind DNA and RNA, and is involved in the regulation of gene expression and controls mRNA export from the nucleus. MATR3 also plays an essential role in Rev-mediated export of HIV-1 mRNA transcript from the nucleus (Kula et al., 2011; Yedavalli and Jeang, 2011), through the interaction with the polypyrimidine tract binding protein and associated splicing factor (PSF) and the viral protein Rev (Zolotukhin et al., 2003). Depletion of MATR3 was demonstrated to inhibit nuclear export of Rev responsive element (RRE)-containing viral RNAs resulting in decreased HIV-1 replication (Sarracino et al., 2018). Resting T cells only express low levels of MATR3 which increases upon activation, indicating a role of this protein in the establishment of latency in resting cells (Sarracino et al., 2018). Since MATR3 is also expressed in monocytes (Ancuta et al., 2009), it can be expected that it is also involved in the posttranscriptional regulation of HIV-1 in these cells.

## Eradication Strategies Targeting the Transcriptionally Latent Reservoir

Even though ART has significantly increased life expectancy of HIV-1 infected individuals, complete eradication of the virus is not achievable without targeting the HIV-1 latent reservoirs. Currently, several therapeutic strategies are explored and early findings suggest that different strategies may be required to eliminate the different cellular viral reservoirs: memory CD4+ T cells which importantly maintain the viral reservoir through homeostatic- or antigen specific proliferation, and long lived cells like macrophages residing in the tissue.

### Latency Reversal

The latent viral reservoir is not recognized by the immune system and therefore these cells are not eliminated. The “shock and kill” strategy aims to induce viral protein production by reactivation of the latent HIV-1 provirus to prompt elimination of the infected cells through recognition by the immune system or cell death as a result of viral replication (Matalon et al., 2011; Badley et al., 2013). The “shock and kill” strategy has been investigated primarily using CD4+ T cells and T cell lines. Histone deacetylases (HDACs), for instance, are important for suppression of HIV-1 proviral DNA expression, and several HDAC inhibitors including SAHA, vorinostat, oxamflatin, metacept-1, and metacept-3 (Shehu-Xhilaga et al., 2009; Badley et al., 2013) have been studied for their ability to reactivate and eliminate latently infected cells. Although HDAC inhibitors have shown activity to reactivate HIV-1 from latent reservoirs in patients on ART, no decline in HIV-1 proviral DNA in CD4+ T cells was observed (Archin et al., 2009a, b, 2012, 2017). In contrast, *in vitro* studies in both the monocytic U1 cell line and monocyte-derived macrophages show that HDAC inhibitors not only reactivate and decrease HIV-1 release, but also degrades the viral particles through the canonical autophagy pathway (Shehu-Xhilaga et al., 2009; Rasmussen et al., 2013; Campbell et al., 2015). Moreover, several other compounds targeting different pathways have also been shown to effectively reactivate latent HIV-1, these include protein kinase C agonists (*in vitro* in both monocytic and T cell lines) (e.g., prostratin and ingenols) (Gulakowski et al., 1997; Korin and Zack, 1999; Warrilow et al., 2006; Colin and Van Lint, 2009; Abreu et al., 2014; Jiang et al., 2014, 2015; Darcis et al., 2015), histone methyltransferases inhibitors (*in vitro* in T cell lines and primary monocytes/macrophages and CD4+ T cells from HIV-1 infected ART treated patients) (Greiner et al., 2005; Miranda et al., 2009; Bernhard et al., 2011; Bouchat et al., 2012), anti-miRNA inhibitors (Zhang, 2009), NF-κB activators (Fernandez et al., 2013; Kumar et al., 2013), and cytokine therapy *in vitro* and *in vivo* in CD4+ T cells as well as in cells of the monocyte/macrophage lineage (Chun et al., 1999; Scripture-Adams et al., 2002; Oguariri et al., 2007). Although the efficacy of the majority of these latency reversing agents (LRAs) has been demonstrated, a major hurdle in this treatment approach is that HIV-1 reactivation only occurs in a small subset of the latently infected cells, which might be overcome at least in part by the use of combinations of these LRAs. Furthermore, targeting of cellular factors and pathways identified to be involved in the regulation of HIV-1 latency at a transcription or post-transcription level, such as DYRK1A, β-catenin/TCF4 and Tat/pTEFb (Kumar et al., 2008; Aljawai et al., 2014; Booiman et al., 2015; Wu et al., 2017; Abner et al., 2018; Table 1) may improve the efficacy of latency reversal. Moreover, LRAs alone are mostly not sufficient to induce cell death and therefore an effective immune response is essential for the elimination of the reactivated cells and this is lacking in the majority of HIV-1 infected patients. Furthermore, HIV-1 infected macrophages can reside in immune privileged compartments like the CNS and can therefore not be reached by cytotoxic T cells (Shan et al., 2012; Badley et al., 2013).

### Apoptosis Inducing Agents

Recently more research is done into the “Prime, Shock, and Kill” method, where latent reservoirs are made sensitive to apoptosis (Prime), followed by reactivation of HIV-1 (Shock) and subsequently leading to cell death of the infected cell (Kill) (Badley et al., 2013). HIV-1 infection of CD4+ T cells and macrophages have been shown to induce an anti-apoptotic gene profile and resistance to apoptosis (Olivares et al., 2009). Several viral proteins have been associated with the resistance to apoptosis (Tanaka et al., 1999; Fernandez Larrosa et al., 2008): Vpr upregulates expression of anti-apoptotic proteins like B-cell lymphoma 2 (Bcl-2) in T cells only, thereby promoting cell survival (Conti et al., 1998). In monocytes/macrophages vpr facilitates HIV-1 replication (Connor et al., 1995). Tat, however, does upregulate Bcl-2 and X-linked inhibitor of apoptosis protein (XIAP) in both monocytes/macrophages and CD4+ T cells (Zhang et al., 2002; Lopez-Huertas et al., 2013); Nef upregulates the anti-apoptotic protein B-cell lymphoma-extra large (Bcl-Xl) in macrophages (Choi and Smithgall, 2004), while nef inactivates the Bcl-2-associated death promoter protein (BAD), a pro-apoptotic Bcl-2 family member, in a phosphoinositide 3-kinase (PI3K)– and p21-activated kinase (PAK)-dependent manner in T cells (Wolf et al., 2001); Furthermore, gp120 has been demonstrated to increase in expression of M-CSF in HIV-1 infected macrophages resulting in the upregulation of anti-apoptotic proteins [e.g., induced myeloid leukemia cell differentiation protein 1 (Mcl-1) and Bcl-2-related protein A1 (Bfl-1)] (Swingler et al., 2007). Therefore, targeting of the HIV-1 induced anti-apoptotic pathways during proviral reactivation strategies may promote cell death of the latently infected cells. Multiple agents targeting the anti-apoptotic pathways including PI3K/Protein kinase B (AKT) inhibitors, Bcl2 inhibitors and second mitochondria-derived activator of caspase (SMAC) mimetics/XIAP inhibitors, are explored for their ability to clear latently infected cells (Busca et al., 2012; Badley et al., 2013; Kim et al., 2018). SMAC mimetics/XIAP inhibitors, for instance, function by sensitizing macrophages to Vpr-induced cell death (Busca et al., 2012). In addition, SMAC mimetics/XIAP inhibitors can trigger transcription of latent proviruses via the non-canonical NF-κB pathway. Furthermore, *in vitro* studies showed that combinations of HDAC inhibitors and SMAC/XIAP mimetics have synergistic activities in T cells (Pache et al., 2015). However, all these compounds do not specifically target the viral reservoir, and targeting of non-HIV-1 infected bystander cells will likely occur especially in ART-treated individuals with increased levels of immune activation, inflammation, and high prevalence of coinfections.

Indeed, an apoptosis resistant gene expression signature was observed in monocytes from HIV-1 infected individuals (Giri et al., 2009). The circulating monocyte population is largely uninfected, which indicates that the anti-apoptotic expression profile may not be due to direct effects of viral infection. Moreover, *in vitro* infection of monocytes/macrophages demonstrated that HIV-1 infection, as well as exposure to HIV-1 or immune modulators increased apoptosis resistance (Giri et al., 2009).

## Concluding Remarks

Despite effective treatment, HIV-1 infected patients are not cured because the virus is not eradicated and latently infected cells persist. The latent viral reservoir mainly resides in resting CD4+ T cells, however, latent HIV-1 proviruses have also been detected in cells of the monocyte/macrophage lineage. HIV-1 infected macrophages can be found in virtually every tissue including the CNS. Viral latency in cells of the monocyte/macrophage lineage can be regulated by diverse molecular mechanisms either at a transcriptional or post-transcriptional level and this is most likely dependent on the activation and differentiation state of these cells. Current eradication strategies rely on reactivation of the latent virus and subsequent kill of the infected cell. The majority of LRAs under study, have been shown to reactivate only small proportions of the latent HIV-1 reservoir, and therefore there is an urgent need for the development of new compounds that target different mechanisms of viral latency both in CD4+ T cells and monocytes/macrophages. Another hurdle is the lack of kill of latently infected cells upon reactivation, which is mainly due to an inefficient antiviral immune response and HIV-1 induced resistance to apoptosis. Apoptosis inducing compounds have been developed for cancer therapy, and their efficacy to specifically induce apoptosis in HIV-1 infected cells is currently under study.

Cure research has mainly focused on CD4+ T cells, because these cells contribute to maintenance pf the viral reservoir through their long lifespan as well as their ability to proliferate under homeostatic conditions or in response to antigens. In contrast, macrophages are terminally differentiated and their contribution to the viral reservoir is mainly dependent on their lifespan and distribution to immune privileged tissues like the CNS. Although, these terminally differentiated latently infected macrophages cannot proliferate, they could be able to spread HIV-1 infection to CD4+ T cells upon reactivation of the virus by LRAs. Therefore, development of eradication strategies combining both novel LRAs and apoptosis inducing agents, targeting either specific cell types or ideally target both latently infected CD4+ T cells and monocytes/macrophages are needed.

## Author Contributions

ZK and NK wrote and edited the review.

## Conflict of Interest

The authors declare that the research was conducted in the absence of any commercial or financial relationships that could be construed as a potential conflict of interest.
